# Comparison of dual‐plane micro‐drop injection of botulinum toxin type A with two traditional methods for treating hypertrophic scars in the tension zone: A long‐term clinical application study

**DOI:** 10.1111/jocd.16401

**Published:** 2024-05-27

**Authors:** Banghong Jiang, Jianting Zhang, Wenjian Yang, Yukun Wang, Sutao Huang, Jingjing Qiu, Bingzhi Bian, Li Zhang, Wenxuan Zu

**Affiliations:** ^1^ Department of Plastic Surgery 1st Affiliated Hospital of Bengbu Medical College Bengbu Anhui province China; ^2^ Bengbu Medical College Bengbu Anhui province China; ^3^ Department of Anatomy Bengbu Medical College Bengbu Anhui China

**Keywords:** botulinum toxin type A, dual‐plane micro‐drop injections, fractional CO_2_ laser, hypertrophic scar, triamcinolone acetonide injection

## Abstract

**Background:**

Patients with hypertrophic scarring tend to experience recurrence after treatment, which often occurs in areas of the body with high skin tension.

**Aims:**

To evaluate better treatments aimed at reducing the risk of scar recurrence in areas of high skin tension.

**Methods:**

Patients were randomly divided into the following three treatment groups: botulinum toxin type A (BTA) via dual‐plane micro‐drop injections, triamcinolone acetonide (TAC) suspension, and CO_2_ via fractional CO_2_ laser. Interventions were implemented in all three groups once a month for three consecutive sessions. After the final treatment, scarring was evaluated at 1, 3, 6, 12, and 24 months using the Patient and Observer Scar Assessment Scale (POSAS).

**Results:**

The 3‐month POSAS score for each scar indicator in the treatment groups was significantly lower than that in the preoperative groups (*p* < 0.001). The scar score in the TAC group decreased at 3 months and increased thereafter. For other groups, the scar score continually decreased at all time points according to the Patient Scar Assessment Scale. Based on the Observer Scar Assessment Scale, the scar score continuously decreased at all time points in the BTA group; in the TAC group, it decreased at 1 month and increased thereafter; and in the CO_2_ group, the scar score decreased at 3 months and subsequently stabilized.

**Conclusions:**

All three treatment methods were effective. However, the BTA group experienced a reduced risk of scar recurrence and maintained long‐term treatment effects.

## 1 INTRODUCTION

Hypertrophic scar (HTS) often forms when an excessive proliferation of collagen fibers occurs during the healing of damaged skin. Approximately 57% of patients develop HTS within 3 months after surgery.^1^ Even after treatment, HTS is prone to recurrence and can increase in size. Several factors influence HTS, among which skin stretch plays an important role. Ogawa^2^ confirmed that decreasing wound stretch can reduce the rate of HTS recurrence.

In daily life, skin tension zones such as the anterior chest, shoulders, back, and suprapubic areas are frequently active, have a high degree of elasticity, and are often affected by mechanical forces closely associated with HTS development.^3^ A continued increase in local tension can indirectly promote the conversion of fibroblasts to myofibroblasts, rendering them more susceptible to HTS.^4^, ^5^ Pathological scar formation is also associated with the overexpression of mechanoreceptors in skin cells and tendons.^6^ Overall, scar treatment in these tension areas should focus on reducing wound tension. Currently, several methods are used to treat HTS, including silicone drugs for external use, drug injection, laser, radiation, freezing, and surgical treatment.^7^, ^8^ Regardless of the management method, HTS remains prone to recurrence, particularly in regions with high mobility and skin tension during daily activities. Resolution to this issue is imperative.

Botulinum toxin type A (BTA), a protein‐like neurotoxin secreted by *Clostridium botulinum*,^9^ was first used as a therapeutic drug by Alan Scott for the clinical treatment of strabismus in 1980.^10^ It can inhibit transforming growth factor β (TGF‐β) synthesis and secretion, reduce the collagen fibers secreted by fibroblasts, and impede excessive scar growth.^11^ BTA can decrease muscle fibrosis by inhibiting the synthesis and secretion of TGF‐β.^12^, ^13^ Additionally, it inhibits the release of acetylcholine from the presynaptic membrane of the neuromuscular junction,^14^ causing muscle paralysis and reducing muscle tone and skin tension thereby hindering pathological scar formation. Theoretically, BTA inhibits scar proliferation in two planes: inside the scar itself and in the muscle beneath it. In contrast to the traditional method of single‐plane injection of drugs into lesions, this study employed a novel approach that combined intralesional and intramuscular micro‐drop injections. Micro‐drop injection is a multi‐point small‐dose injection of BTA into the dermis or between the dermis and the muscle layer. A small dose prevents diffusion into deeper muscles, thus preserving muscle function and avoiding adverse reactions.^15^ In the Kim and Khatery trial,^16^, ^17^ the follow‐up period was a maximum of 6 months; however, to explore the long‐term BTA effects on HTS, this study set a follow‐up period of up to 2 years.

Our study was conducted to explore the feasibility and long‐term effect of dual‐plane micro‐drop BTA injection on HTS compared to that of two traditional treatments, namely, triamcinolone acetonide (TAC) injection and fractional CO_2_ laser. The Patient and Observer Scar Assessment Scale (POSAS) was used for assessment during the 2‐year follow‐up.

## 2 MATERIALS AND METHODS

### 2.1 Patients

This randomized controlled clinical trial was conducted after obtaining approval from the Hospital Ethics Committee for the Application of Clinical Technology on January 1, 2022 (approval number 2022025). Written informed consent was obtained from all participants before treatment. The experimental registration number, ChiCTR2300073981, was obtained from the Chinese Clinical Trial Registry (chictr.org.cn).

A total of 45 patients with HTS aged 8–68 years were selected according to the inclusion criteria (demographic data is shown in Table 1). Patients were randomly divided into three groups using the Excel random number table method, with 15 individuals per group. Each group received the appropriate intervention once a month for three consecutive sessions. The POSAS was used to assess progress continuously for 2 years, and results were recorded. The scoring scale was drawn using Procreate Prism 9 (Tasmania, Australia), and the data for each group were compared.

**TABLE 1:** Patient demographic data.

	BTA	TAC	*p* (BTA vs. TAC)	CO_2_	*p* (BTA vs. CO_2_)
Age at operation	32.73 ± 16.80	30.00 ± 14.61	0.648	32.13 ± 17.28	0.920
Sex (male:female)	(5:10)	(5:10)	1.000	(6:9)	1.000
Location (face and neck:limbs:torso)	(4:8:3)	(4:7:4)	0.901	(3:8:4)	0.867

The inclusion criteria were as follows: (1) satisfied the HTS diagnostic criteria, (2) presence of scars in active areas with tension, (3) no prior treatment, (4) no history of muscle weakness or botulinum toxin allergy, and (5) willing to accept treatment with follow‐up.

The exclusion criteria were as follows: (1) the presence of atrophic scarring, (2) pregnant and lactating women, and (3) comorbid underlying conditions.

### 2.2 Treatment procedure

Each group was anesthetized by injecting 2% lidocaine from the edge into the deeper part of the lesion to prevent secondary keloid formation.

In the BTA group, with the needle tip adjusted to scar thickness (1–4 mm), microdroplets of BTA (Hengli, Lanzhou Biotechnology Development Co. Ltd., China) were injected into the scar lesion at 0.5‐cm intervals, and 1 − 2 U were subsequently injected at each point into the subcutaneous muscle under ultrasound guidance (Figure 1). It was important to consider the injection dose according to the thickness and location of the muscle, especially facial scarring needed to be injected symmetrically on both the left and right sides and controlled the dose of BTA to prevent unilateral excessive injection, which could result in significant asymmetry in facial expression or muscle thickness. In the TAC group, each scar lesion was injected with a TAC suspension (Haipu, Shanghai Xudong Haipu Pharmaceutical Co. Ltd., China) at 1‐cm intervals until the scar turned white. The CO_2_ group used a 10 600‐nm ablative fractional CO_2_ laser with a power setting of 30–60 J, interval of 6.3, and 9% coverage to sweep the scar tissue. All three group interventions were implemented once a month for three consecutive sessions. After the final treatment, the POSAS was used to evaluate scarring in the three groups at 1, 3, 6, 12, and 24 months.

**FIGURE 1:**
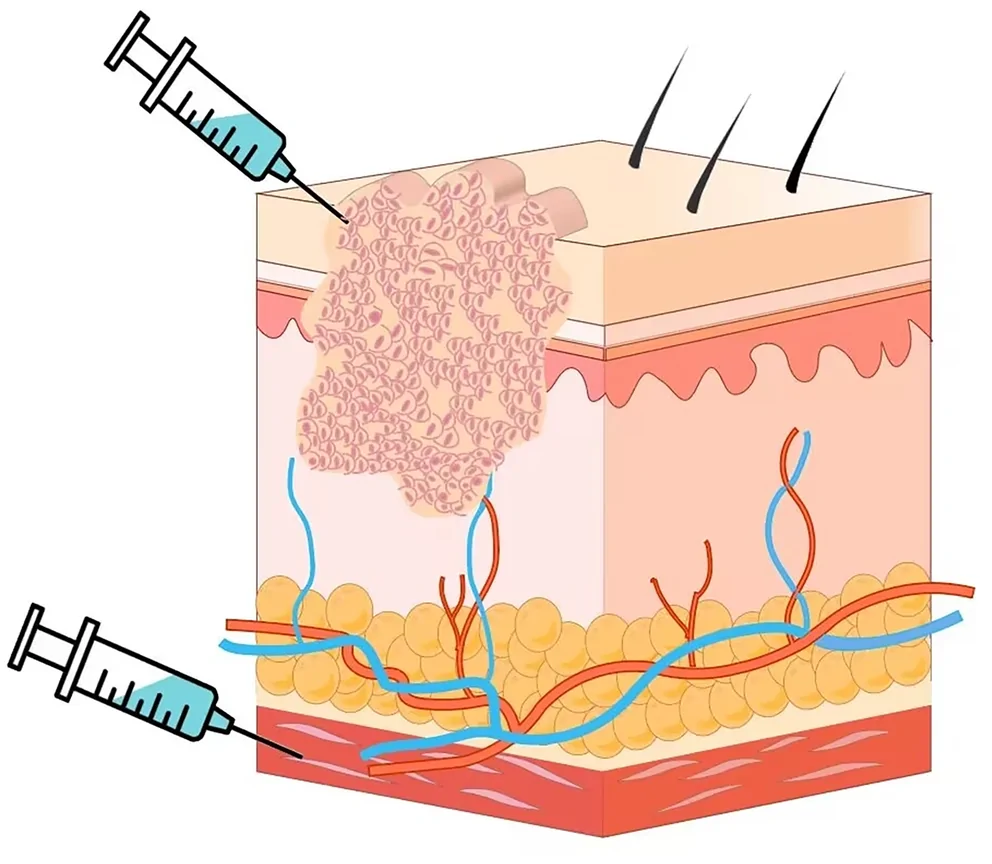
Schematic diagram of the dual‐plane micro‐drop injection of BTA for HTS treatment.

### 2.3 Evaluation methods

Indicators such as pain, pruritus, thickness, surface roughness, pliability, and color were assessed using the Patient Scar Assessment Scale (P‐SAS). The Observer Scar Assessment Scale (O‐SAS) was used to evaluate changes in the following indicators: surface area, thickness, surface roughness, pigmentation, vascularization, and pliability. Capillary refill during release was assessed by pressing the sides around each scar and assessing vascularity. Each indicator was rated on a scale of 1 to 10, with 1 being the best and 10 being the worst. The preoperative scar scores were all rated as 10. The follow‐up results were calculated by averaging the sum of all index scores.

### 2.4 Statistical analysis

All data were initially entered into an Excel database (Microsoft, Redmond, WA, USA) and analyzed using GraphPad Prism 9 (California, USA). Indicators of the POSAS were compared before and after treatment using a one‐way ANOVA with significance set at *p* < 0.5. The effects of the three different therapeutic drugs on scar scores were compared using a two‐way ANOVA.

## 3 RESULTS

Three months after the interventions, the POSAS scores of all scar indicators in the three treatment groups were significantly lower than those in the preoperative groups (Figure 2A) (*p* < 0.001). The BTA group had the lowest scores for pain and surface roughness; the TAC group had overall low scores, except for vascularization; the overall scores in the CO_2_ group were high except for thickness, vascularization, and flexibility.

**FIGURE 2:**
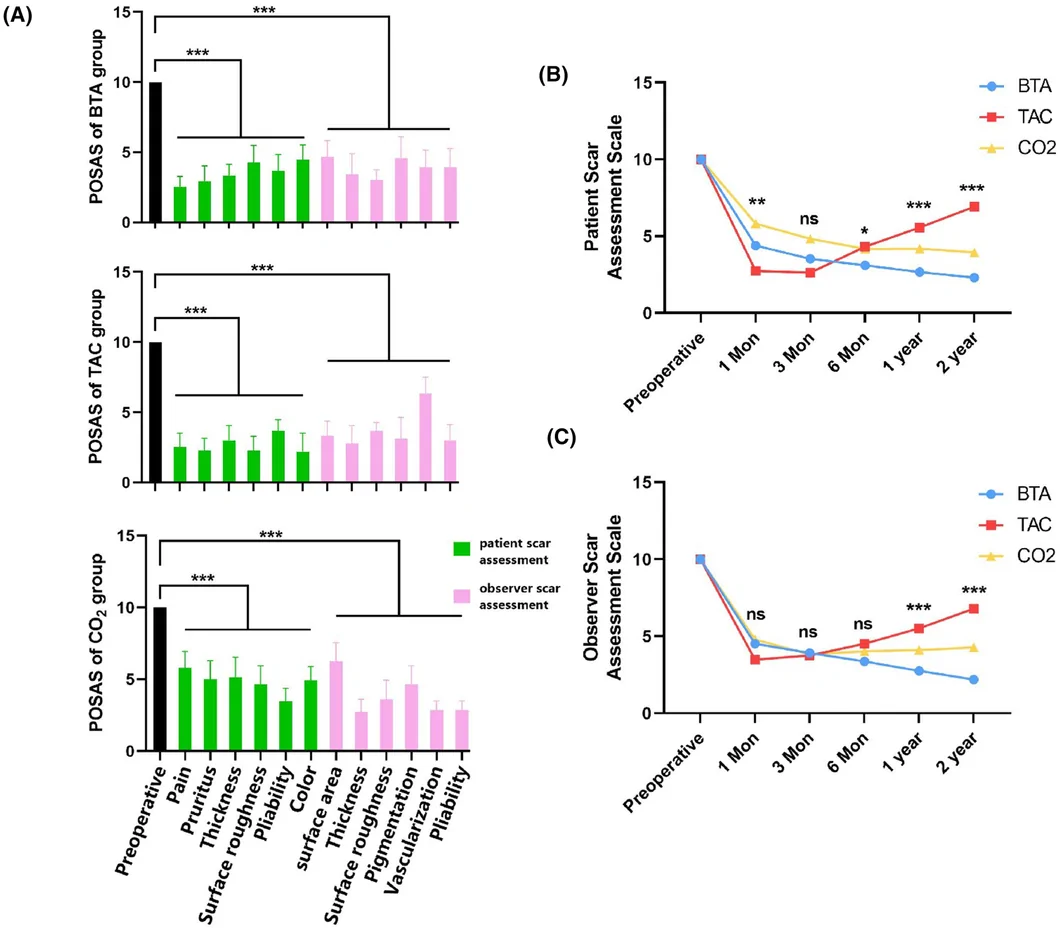
Results of the scar treatment according to the Patient and Observer Scar Assessment Scale (POSAS) (A) At 3 months of follow‐up, the POSAS score for each scar indicator in the treatment groups was significantly lower than that in the preoperative group. BTA vs. TAC, BTA versus. CO_2_; ns *p* > 0.05, * *p* < 0.05, ** *p* < 0.01, *** *p* < 0.001 compared with preoperative values. Results of scar treatment according to the Patient Scar Assessment Scale (P‐SAS) (B) the P‐SAS scores in the TAC group decreased at 3 months of follow‐up and increased thereafter. In other groups, a continuous decrease occurred at all time points. BTA versus. TAC, BTA versus. CO_2_; ns *p* > 0.05, * *p* < 0.05, ** *p* < 0.01, *** *p* < 0.001 compared with preoperative values. Results of scar treatment according to the Observer Scar Assessment Scale (O‐SAS) (C) In the BTA group, a continuous decrease occurred at all time points; the O‐SAS score in the TAC group decreased at 1 month of follow‐up and increased thereafter; the scar score in the CO_2_ group decreased at 3 months of follow‐up and stabilized thereafter. BTA vs. TAC, BTA vs. CO_2_; ns *p* > 0.05, * *p* < 0.05, ** *p* < 0.01, *** *p* < 0.001 compared with preoperative values. The scales are scored 1–10: a score of 1 indicates that the scarring features are close to normal skin, and higher scores indicate more severe scarring. BTA: Botulinum toxin type A, TAC: triamcinolone acetonide.

According to the P‐SAS (Figure 2B), the TAC group showed an increase in scar scores at the three‐month postoperative follow‐up, and then a further increase after the six‐month postoperative follow‐up, which indicates a worsening of the scars. Scar scores continued to decrease at follow‐up in the CO_2_ and BTA groups, and the BTA group scores were consistently lower than those of the CO_2_ group.

At the one‐month follow‐up, the treatment scores for all three groups were significantly lower than those before treatment, with the TAC group having the lowest scores (*p* < 0.01).

At the three‐month follow‐up, the scores of the three groups continued to decline (*p* > 0.05). At the six‐month postoperative follow‐up, the scar scores in the TAC group were higher than those in the previous measurements, whereas the scores in the other two groups continued to decrease. The BTA group had the lowest scores (*p* < 0.05).

During follow‐up assessments in the first and second years after surgery, the TAC group consistently had higher scar scores than the other two groups. In contrast, the remaining two groups showed a downward trend in scar scores, with the lowest scores observed in the BTA group (*p* < 0.001).

According to the O‐SAS (Figure 2C), the TAC group showed an increase in scar scores at the third month of follow‐up; the CO_2_ group scores increased slightly after the third month of follow‐up, and the scores continued to decrease in the BTA group at follow‐up.

At the one‐month postoperative follow‐up, the scar scores for all three groups were lower than their preoperative scores. The TAC group had the lowest scores, and the other two groups had similar scores (*p* > 0.05).

At the three‐month follow‐up, the TAC group scores were higher than those at the first month follow‐up, whereas the scores of the CO_2_ and BTA groups decreased. However, the three groups had similar follow‐up scores (*p* > 0.05).

At the six‐month postoperative follow‐up, the scar scores of the TAC group remained elevated, those of the CO_2_ group showed no change from previous scores, and the scores of the BTA group decreased (*p* > 0.05).

At the one‐year postoperative follow‐up visit, the TAC group scores remained elevated, those of the CO_2_ group were slightly higher, and the BTA group scores continued to decrease (*p* < 0.001).

At the second‐year postoperative follow‐up, the scar scores in the TAC group were similar to the preoperative scores. The scores of the CO_2_ group were slightly higher than previous scores, whereas the scores of the BTA group continued to decrease (*p* < 0.001).

Patient satisfaction is indirectly related to scar scores as shown in Figure 2B. Lower scar scores indicate better scar recovery and higher patient satisfaction. The patients in the TAC group had the lowest scar scores and highest satisfaction during the first 3 months of follow‐up, but satisfaction decreased after the sixth month. Patient satisfaction in the CO_2_ and BTA groups continued to increase during follow‐up, with the BTA group consistently having higher satisfaction than the CO_2_ group. Patient satisfaction in the BTA group was the highest at 6 months of follow‐up.

Comparison images of patients treated with BTA dual‐plane micro‐drop injection are shown in Figures 3, 4, and 5. The photos of patient treatments in the TAC and CO_2_ groups have been uploaded as Figures S1 and S2.

**FIGURE 3:**
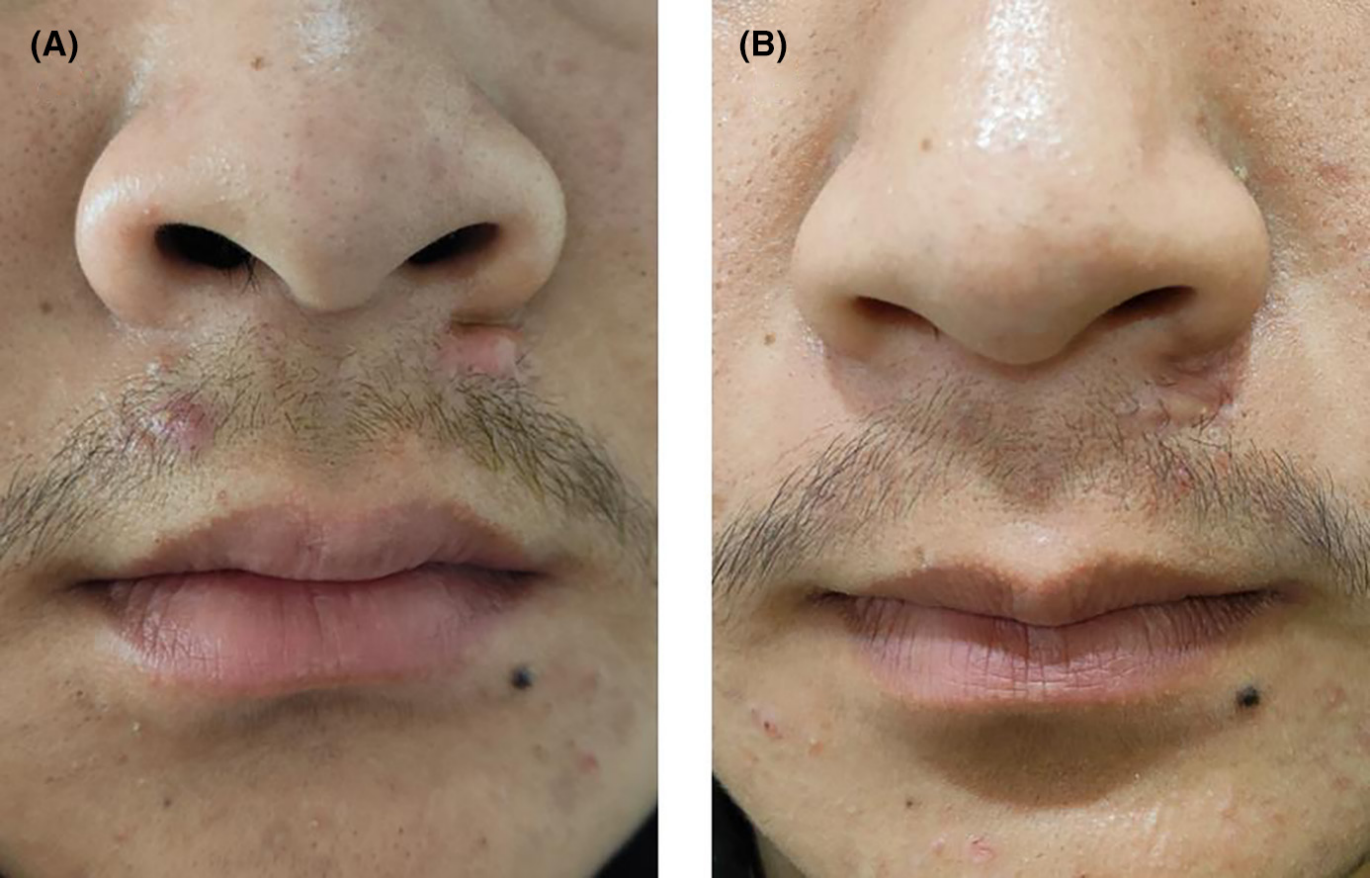
Photographic imaging of Patient's face HTS before and after designated treatments:before (A) and after (B).

**FIGURE 4:**
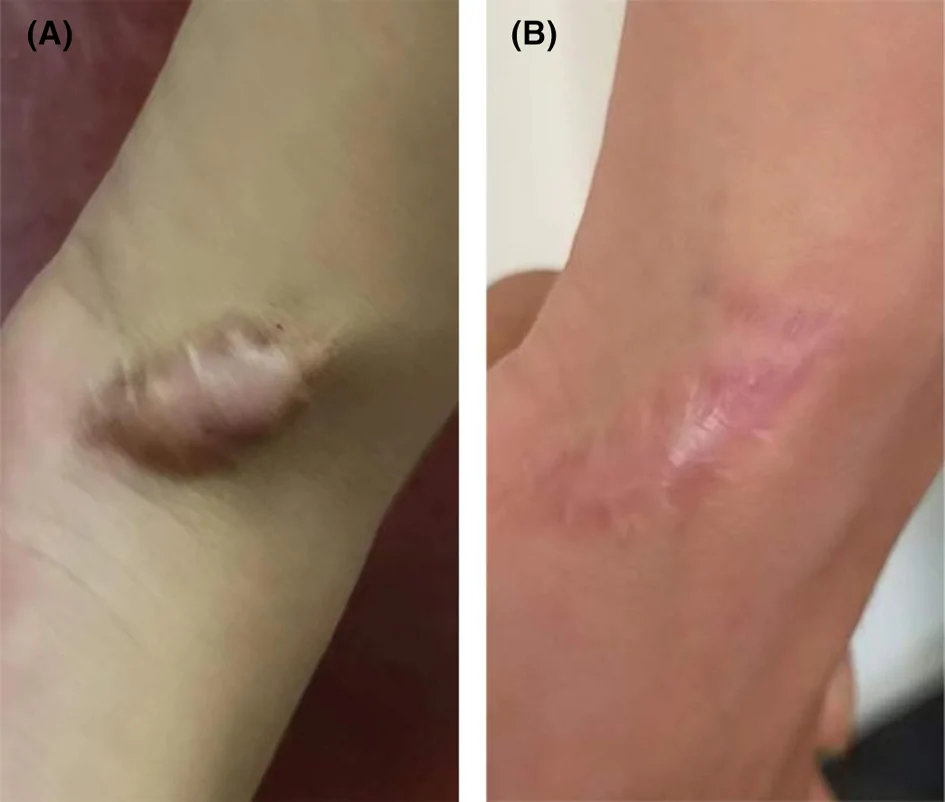
Photographic imaging of Patient's wrist HTS before and after designated treatments:before (A) and after (B).

**FIGURE 5:**
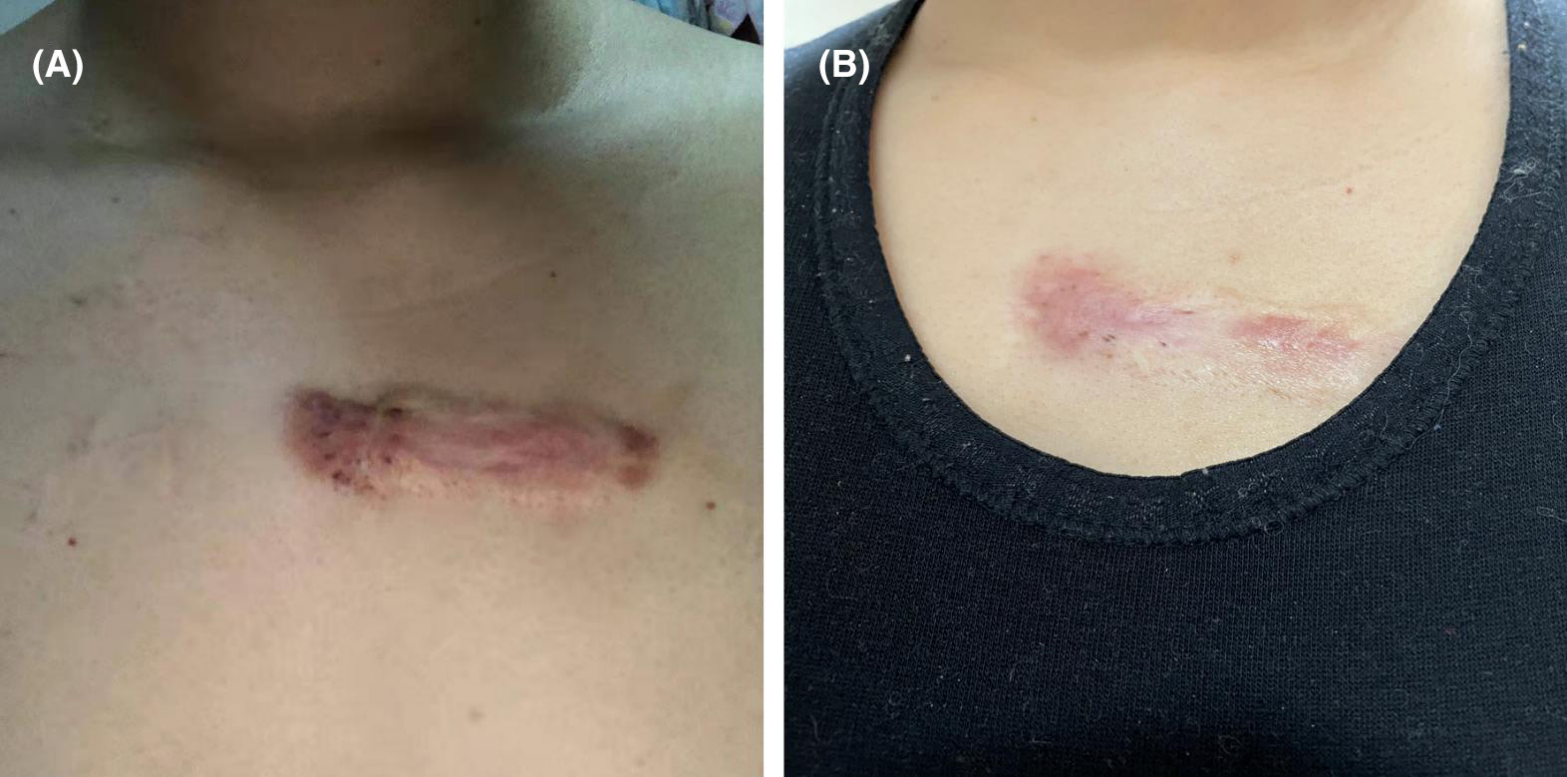
Photographic imaging of Patient's precordial region HTS before and after designated treatments:before (A) and after (B).

## 4 DISCUSSION

HTS commonly occurs in areas of high skin tension, such as the shoulders, chest, and limb joints. Excessive tension can cause increased production of extracellular matrix, leading to the formation and exacerbation of HTS. According to Ogawa et al., skin tension is a crucial factor in triggering scar formation, and reducing skin tension around wounds or scars is an effective measure for preventing and treating pathological scars.^18^ Tension zone scarring is often mechanically irritated and prone to recurrence after surgical treatment, reducing patients' confidence in the treatment and exacerbating pain.

The treatment was effective in all three groups at the third postoperative month of follow‐up, and the TAC group showed the most significant decrease in all indicators and the best scar recovery. According to the P‐ASA and O‐ASA, the scar scores in the TAC group showed a rapid decrease and then an increase, with the turning points being the sixth and the third month of postoperative follow‐up, respectively, which implies that TAC can only inhibit scar growth rapidly in the short term, however, the scar scores in the CO2 group and the BTA group were decreasing throughout the follow‐up period, and the decreasing trend was more pronounced in the BTA group in particular, which suggests that both the CO2 laser and the dual‐plane micro‐drop injection of BTA are able to inhibit scar proliferation for a long period of time, with the BTA being more potent in long‐term inhibition.

BTA can inhibit the secretion of TGF‐β by fibroblasts, the conversion of fibroblasts to myofibroblasts, and the secretion of collagen fibers. Additionally, it can prevent excessive deposition of extracellular matrix, disordered arrangement of collagen, and excessive proliferation of scars. BTA can reduce collagen secretion by inhibiting the SP‐NK1R signaling pathway.^19^ Compared with the usual intralesional injection, this study added a new plane of intramuscular injection, in which BTA can reduce scar tension and inhibit scar growth by inhibiting acetylcholine release at the neuromuscular junction. Moreover, the effect of changes in the mechanical environment on HTS may be related to the mechanical cation channel of piezoelectric 1.^20^ In addition, the use of the micro‐drop injection technique in this study resulted in a smaller single injection dose and a lower drug concentration than those used in the traditional injection technique. This technique avoids complications such as peripheral muscle paralysis associated with traditional single high‐dose injections and offers a more superficial injection level, making it safer and more precise.^21^ Dual‐plane injection maximizes the efficacy of BTA's anti‐fibrotic and wound tension‐reducing benefits, which are the technical highlights of this study. Compared to the six‐month follow‐up time in Khatery's study,^17^ this study set a follow‐up period of up to 2 years, and the enduring inhibitory effect of botulinum toxin on HTS was clearly observed.

TAC is widely acknowledged as the first line of treatment for HTS and has often been compared to newer approaches. Most of the known effects of corticosteroids are thought to be primarily due to suppression of the inflammatory response and secondarily due to diminished collagen synthesis, inhibition of fibroblast growth, and enhanced collagen degeneration.^22^ Multiple injections are necessary to avoid recurrence because TAC only provides short‐term inhibition. Scars in tension zones are often caused by mechanical forces and are more likely to recur. Prolonged use of steroids may result in advanced effects such as skin atrophy, telangiectasia, ulceration, and hypopigmentation.^23^ Additionally, the safety and efficacy of TAC injections as monotherapy may vary among individuals. One meta‐analysis of its use showed a 50%–100% range of regression rates and a 33%–50% recurrence rate.^24^

Fractional CO_2_ laser treatment is also one of the commonly used treatment methods in clinical practice. Its mechanism of action involves using a laser with a diameter of <500 microns to irradiate scar tissue in a fractional pattern, causing deep penetration, forming columnar damage between scar tissues, and stimulating the normal cells between columns to play a functional role in tissue repair and remodeling.^25^ This method provides significant scar treatment, long‐lasting efficacy, and low adverse effects, but it causes extensive skin damage and requires a long recovery time.^26^

Patients in the BTA group demonstrated a stable treatment effect and a low scar recurrence rate at the 2‐year follow‐up, thus exhibiting a greatly improved quality of life. Patients in the TAC group needed several injections to maintain suppression effects, and a progressive decline in patient satisfaction was observed. The CO_2_ group showed long‐lasting efficacy and few adverse effects; however, it caused extensive skin damage and required a long recovery time.

The limitation of this study is the small sample size, with only 15 patients in each group. Meanwhile, the relaxation effect of botulinum toxin on mechanical force around HTS needs further experiments to be revealed. In the future, we will develop personalized combined treatment plans for patients based on the strengths of each method. We hope that the dual‐plane micro‐drop injection method will provide a reference for other investigators conducting studies on BTA for scars, with the goal of reducing the postoperative recurrence rate of HTS in the tension zone.

## 5 CONCLUSIONS

The BTA group had the best prognosis and prominent long‐lasting inhibitory effects, which reduced the recurrence rate of scarring. The TAC group showed the best immediate treatment effect; however, the risk of recurrence was high. The CO2 group exhibited moderate long‐lasting inhibition efficacy.

## AUTHOR CONTRIBUTIONS

Banghong Jiang, Yukun Wang and Li Zhang are responsible for patient recruitment.

Banghong Jiang and Wenjian Yang have made substantial contributions to acquisition of data, or analysis and interpretation of data. Banghong Jiang and Jianting Zhang have been involved in drafting the manuscript or revising it critically for important intellectual content and given final approval of the version to be published. Sutao Huang, Jingjing Qiu, Bingzhi Bian and Wenxuan Zu have helped conceptualize, search for literature, and submit articles. Each author has participated sufficiently in the work to take public responsibility for appropriate portions of the content and agreed to be accountable for all aspects of the work in ensuring that questions related to the accuracy or integrity of any part of the work are appropriately investigated and resolved.

## FUNDING INFORMATION

The work was supported by the following projects and organizations: Anhui university provincial natural science research project (KJ2021A0772; 2022AH051451); National College Students Innovation and Entrepreneurship Training Program (NO.202210367015); Anhui Province College Students Innovation and Entrepreneurship Training Program (NO.S202210367003; S202310367004). National Natural Youth Foundation Incubation Program of Bengbu Medical College (2023byfy009).

## CONFLICT OF INTEREST STATEMENT

The authors declare that they have no conflicts of interest to disclose.

## ETHICS STATEMENT

The study was approved by the Ethics Committee for the Application of Clinical Technology of the First Affiliated Hospital of Bengbu Medical College on January 1, 2022, with approval number 2022025.

All procedures performed in studies involving human participants were in accordance with the ethical standards of the institutional and/or national research committee and with the 1964 Helsinki declaration and its later amendments or comparable ethical standards.

## Supporting information


Figure S1.

Figure S2.

## Data Availability

The data that support the findings of this study are available from the corresponding author upon reasonable request.

## References

[1] Mahdavian Delavary B , van der Veer WM , Ferreira JA , Niessen FB . Formation of hypertrophic scars: evolution and susceptibility. J Plast Surg Hand Surg. 2012;46(2):95‐101. 10.3109/2000656X.2012.669184 22471257

[2] Ogawa R , Akaishi S , Huang C , et al. Clinical applications of basic research that shows reducing skin tension could prevent and treat abnormal scarring: the importance of fascial/subcutaneous tensile reduction sutures and flap surgery for keloid and hypertrophic scar reconstruction. J Nippon Med Sch. 2011;78(2):68‐76. 10.1272/jnms.78.68 21551963

[3] Sano H , Hokazono Y , Ogawa R . Distensibility and gross elasticity of the skin at various body sites and association with pathological scarring: a case study. J Clin Aesthet Dermatol. 2018;11(6):15‐18. PMC6011867 29942420

[4] Harn HI , Ogawa R , Hsu CK , Hughes MW , Tang MJ , Chuong CM . The tension biology of wound healing. Exp Dermatol. 2019;28(4):464‐471. 10.1111/exd.13460 29105155

[5] Butzelaar L , Ulrich MM , Mink van der Molen AB , Niessen FB , Beelen RH . Currently known risk factors for hypertrophic skin scarring: a review. J Plast Reconstr Aesthet Surg. 2016;69(2):163‐169. 10.1016/j.bjps.2015.11.015 26776348

[6] Ogawa R . Keloid and hypertrophic scarring may result from a mechanoreceptor or mechanosensitive nociceptor disorder. Med Hypotheses. 2008;71(4):493‐500. 10.1016/j.mehy.2008.05.020 18614294

[7] Arno AI , Gauglitz GG , Barret JP , Jeschke MG . Up‐to‐date approach to manage keloids and hypertrophic scars: a useful guide. Burns: Journal of the International Society for Burn Injuries. 2014;40(7):1255‐1266. 10.1016/j.burns.2014.02.011 PMC4186912 24767715

[8] Poetschke J , Gauglitz GG . Current options for the treatment of pathological scarring. J Dtsch Dermatol Ges. 2016;14(5):467‐477. 10.1111/ddg.13027 27119465

[9] Pirazzini M , Rossetto O , Eleopra R , Montecucco C . Botulinum neurotoxins: biology, pharmacology, and toxicology. Pharmacol Rev. 2017;69(2):200‐235. 10.1124/pr.116.012658 PMC5394922 28356439

[10] Scott AB . Botulinum toxin injection into extraocular muscles as an alternative to strabismus surgery. Ophthalmology. 1980;87(10):1044‐1049. 10.1016/s0161-6420(80)35127-0 7243198

[11] Zhang X , Lan D , Ning S , Jia H , Yu S . Botulinum toxin type a prevents the phenotypic transformation of fibroblasts induced by TGF‐β1 via the PTEN/PI3K/Akt signaling pathway. Int J Mol Med. 2019;44(2):661‐671. 10.3892/ijmm.2019.4226 PMC6605626 31173164

[12] Jiang B , Zu W , Xu J , et al. Botulinum toxin type a relieves sternocleidomastoid muscle fibrosis in congenital muscular torticollis. Int J Biol Macromol. 2018;112:1014‐1020. 10.1016/j.ijbiomac.2018.02.077 29447964

[13] Li YH , Yang J , Zheng Z , Hu DH , Wang ZD . Botulinum toxin type a attenuates hypertrophic scar formation via the inhibition of TGF‐β1/Smad and ERK pathways. J Cosmet Dermatol. 2021;20(5):1374‐1380. 10.1111/jocd.13842 33185943

[14] Matak I , Lacković Z . Botulinum neurotoxin type a: actions beyond SNAP‐25? Toxicology. 2015;335:79‐84. 10.1016/j.tox.2015.07.003 26169827

[15] Ng ZY , Lellouch AG . Use of micro botulinum toxin for a face‐lifting effect: a systematic review. Dermatol Surg. 2022;48(8):849‐854. 10.1097/DSS.0000000000003483 35560135

[16] Kim SH , Lee SJ , Lee JW , Jeong HS , Suh IS . Clinical trial to evaluate the efficacy of botulinum toxin type a injection for reducing scars in patients with forehead laceration: a double‐blinded, randomized controlled study. Medicine. 2019;98(34):e16952. 10.1097/MD.0000000000016952 PMC6716761 31441893

[17] Khatery BHM , Hussein HA , Abd‐El‐Raheem TA , El Hanbuli HM , Yassen NN . Assessment of intralesional injection of botulinum toxin type a in hypertrophic scars and keloids: clinical and pathological study. Dermatol Ther. 2022;35(10):e15748. 10.1111/dth.15748 36190006

[18] Ogawa R . Mechanobiology of cutaneous scarring. In: Téot L , Mustoe TA , Middelkoop E , Gauglitz GG , eds. Textbook on Scar Management: State of the art Management and Emerging Technologies. Springer; 2020:11‐18. 36351054

[19] Zhang S , Li K , Yu Z , et al. Dramatic effect of botulinum toxin type a on hypertrophic scar: a promising therapeutic drug and its mechanism through the SP‐NK1R pathway in cutaneous neurogenic inflammation. Front Med. 2022;9:820817. 10.3389/fmed.2022.820817 PMC8927735 35308522

[20] He J , Fang B , Shan S , et al. Mechanical stretch promotes hypertrophic scar formation through mechanically activated cation channel Piezo1. Cell Death Dis. 2021;12(3):226. 10.1038/s41419-021-03481-6 PMC7921104 33649312

[21] Li SL , Wang YQ , Song T , Hu JT . Microdrop Botox technique for dynamic wrinkles in the upper face. Aesthetic Plast Surg. 2022;46:2625‐2626. 10.1007/s00266-022-02789-8 35089387

[22] Reish RG , Eriksson E . Scar treatments: preclinical and clinical studies. J Am Coll Surg. 2008;206(4):719‐730. 10.1016/j.jamcollsurg.2007.11.022 18387479

[23] Deshmukh NS , Belgaumkar VA , Mhaske CB , Doshi BR . Intralesional drug therapy in dermatology. Indian J Dermatol Venereol Leprol. 2017;83(1):127‐132. 10.4103/0378-6323.190870 27647355

[24] Morelli Coppola M , Salzillo R , Segreto F , Persichetti P . Triamcinolone acetonide intralesional injection for the treatment of keloid scars: patient selection and perspectives. Clin Cosmet Investig Dermatol. 2018;11:387‐396. 10.2147/CCID.S133672 PMC6063260 30087573

[25] Xu Y , Deng Y . Ablative fractional CO2 laser for facial atrophic acne scars. Facial Plast Surg. 2018;34(2):205‐219. 10.1055/s-0037-1606096 29304516

[26] Peng W , Zhang X , Kong X , Shi K . The efficacy and safety of fractional CO(2) laser therapy in the treatment of burn scars: a meta‐analysis. A meta‐analysis.Burns. 2021;47(7):1469‐1477. 10.1016/j.burns.2021.08.010 34493423

